# Is the quality of public health facilities always worse compared to private health facilities: Association between birthplace on neonatal deaths in the Indian states

**DOI:** 10.1371/journal.pone.0296057

**Published:** 2023-12-27

**Authors:** Priyanka Dixit, Thiagarajan Sundararaman, Shiva Halli

**Affiliations:** 1 School of Health Systems Studies, Tata Institute of Social Sciences, Mumbai, India; 2 Adjunct Faculty, IIT Madras, Chennai, India; 3 Department of Community Health Sciences Faculty of Medicine, University of Manitoba, Manitoba, Canada; Indian Institute of Dalit Studies (IIDS), INDIA

## Abstract

**Background:**

The role of place of delivery on the neonatal health outcomes are very crucial. Although the quality of care is being improved, there is no consensus about who is the better healthcare provider in low and middle-income countries (LMICs), public or private facilities. The aim of this study is to assess the differentials in neonatal mortality by the type of healthcare providers in India and its states.

**Methods:**

We used the data from the fourth wave of the National Family Health Survey 2015–16 (NFHS-4). Information on 259,627 live births to women within the five years preceding the survey was examined. Neonatal mortality rates for state and national levels were calculated using DHS methodology. Multi-variate logistics regression was performed to find the effect of birthplace on neonatal deaths. Propensity score matching (PSM) was used to evaluate the relationship between place of delivery and neonatal deaths to account for the bias attributable to observable covariates.

**Results:**

The rise in parity of the women and purchasing power influences the choice of healthcare providers. Increased neonatal mortality was found in private hospital delivery compared to public hospitals in Punjab, Rajasthan, Chhattisgarh, Madhya Pradesh, Bihar, Jharkhand, Odisha, Goa, Maharashtra, Andhra Pradesh and Karnataka states using propensity score matching analysis. However, analysis on the standard of pre-natal and post-natal care indicates that private hospitals generally outperformed public hospitals.

**Conclusions:**

The study observed a significant variation in neonatal mortality among public and private health care systems in India. Findings of the study urges that more attention be paid to the improve care at the place of delivery to improve neonatal health. There is a need of strengthened national health policy and public-private partnerships in order to improve maternal and child health care in both private and public health facilities.

## Background

According to 2016 estimates, India ranks 12^th^ out of 52 low-middle-income nations with the highest infant mortality rates, with over six lakh children dying during the first month of life [1]. Though under-five mortality has decreased by roughly 30% since 2012, new-born deaths have decreased by 26% and neonatal fatalities by just 22% during the same period. As a result, almost half (47%) of all deaths in children under five occur within the first month of life [2]. Reducing new-born mortality is thus the primary goal of international and national public health policy.

New-born deaths have been strongly associated with delivery-related complications such as intrapartum asphyxia, birth trauma, and premature birth [3, 4]. The main strategy for ensuring this determinant of neonatal mortality has been universalising access to skilled birth assistance [5, 6] Institutional delivery has been touted as one of the most effective ways of ensuring access to skilled birth assistance and guaranteeing the quality and continuity of care required to improve maternal and neonatal survival. In India, Janani Suraksha Yojana (JSY), a conditional cash transfer scheme provides demand-side financing of about Rs. 1200 to encourage families to opt for institutional delivery. JSY has led to a major acceleration in institutional delivery, the rates increasing from less than 38.7% in 2005–06 (NFHS-3) to 88.6% in 2019–21 (NFHS-5). One rationale behind introducing demand-side financing was that families would be free to choose between public and private providers, especially in the states where the public sector was weak. In practice, partly due to the terms of implementation, the major increase in institutional delivery was seen in the public sector.

Since 2008, the Government of India has implemented several publicly sponsored insurance programmes that cover both normal and C-section births. The Pradhan Mantri Jan Arogya Yojana (PM-JAY) programme has raised hospital reimbursement to over Rs. 6000 for normal deliveries and more for complex and C-sections. Several studies, however, have questioned the efficiency of increased institutional births in lowering poor outcomes for mothers and children in developing nations [7, 8].

The major cause for this has been linked to poor quality in public healthcare institutions. This has prompted some to doubt the necessity of the forced move to institutional delivery. Others have argued for making it more lucrative for the private sector to participate in providing care, and the inclusion of PMJAY in more flexible terms is regarded as a reaction to this. The quality of care offered by public and commercial providers in low and middle-income nations such as India has long been a contentious issue in the global health arena.

In India, it is often assumed that the quality of care offered in the private sector is superior, competent, and responsible to that provided in the public sector. The argument between the public and private sectors is primarily divided into two groups, with the one demanding the provision of universal state-based healthcare and the second emphasising private sector treatment, particularly in places where the public sector has not been very effective. Many impoverished individuals seek care from private healthcare providers, according to proponents of the private sector [9] and this sector is more competent and attentive to patient demand owing to greater market competition [10]. In contrast, public sector proponents highlight discrepancies in access to health care in the private sector as a result of high healthcare costs. A thorough examination of research papers on the performance of the public and private sectors in poor and middle-income countries, including India, discovered that the concept that the private sector is more responsible, efficient, or medically effective than the public sector is a prejudiced assumption. However, the public sector was deemed to be weak in punctuality and patient friendliness [11].

Therefore, the main goal of this study is to evaluate the association between type of healthcare facilities (public and private health facility) and neonatal mortality in India and its states. With the help of these relation study wanted to highlight the state-level quality of the public and private facilities for pregnancy and infant care. The association between neonatal mortality and healthcare provision in public and private health facilities can offer insights into the quality of Maternal and Child Health (MCH) care offered by each sector. When comparing neonatal mortality rates, lower rates in either public or private hospitals may suggest superior MCH care in that particular sector. However, to accurately assess the effect of the place of delivery on neonatal mortality, study employed propensity score matching (PSM) to control for confounding factors such as socio-economic status and demographic influences. This rigorous approach isolates and examine the true effect of the place of delivery on neonatal mortality, considering factors beyond the hospital’s control that might also influence outcomes. By employing PSM, study aims to gain a clearer understanding of the actual association between the place of delivery and neonatal mortality rates, enabling us to make more informed conclusions about the quality of MCH care provided by public and private hospitals. Additionally, the use of antenatal care components and postnatal care of the new-born was also examined to support our findings.

## Materials and methods

### Data sources: Sample size and design

The current study makes use of data from the fourth wave of the National Family Health Survey 2015–16 (NFHS-4). The NFHS is a large-scale, multi-stage survey of a representative sample of Indian households. It is comparable to demographic and health surveys undertaken in many nations worldwide. NFHS-4 employed a stratified, multistage cluster sampling procedure to create a nationally representative sample of households. To choose households from all 32 states and union territories, the two-stage probability proportional to size (PPS) sample approach was used in rural regions, while the three-stage PPS sampling method was used in urban areas. NFHS-4 offers demographic, health, and government programme information for India and each state/union territory.

The survey, which gathered information from 601, 509 homes, 699,686 women, and 103, 525 men, gives district-level figures for several crucial variables for the first time. The current study makes use of data from births that occurred five years before the survey date. Nationally, a total of 259,627 children were born to 190,898 women in the five years preceding the survey date. More information regarding sampling design, survey team training, and survey administration are there in the country-level reports issued by ORC Macro International [12].

### Measurements

#### Treatment variable

This study aims to investigate the association between place of delivery and neonatal deaths in India and its states. Mothers who gave birth in the five years preceding the survey were asked about the place of delivery in NFHS-4. For all successive births in the five years preceding the survey, information on the place of delivery is available. Births in health facilities such as governmental institutions, Non-Governmental Organization (NGO) trusts, and private hospitals are referred to as ’institutional delivery,’ but births at one’s own house, parents’ homes, or other people’s homes are referred to as ’home delivery.’ The study seeks to compare infant deaths in public and private health facilities which indicates the quality of health facilities.

Institutional delivery is divided into public and private institutes. Based on the location of the live birth, a dichotomous treatment variable was constructed. If a live birth occurred in a government hospital, government dispensary, urban health centre, urban family welfare clinic, community health centre, or primary health centre, it was considered ’public’ and given code "0," and ’private’ and given code "1" if the delivery occurred in a private hospital, private clinic, non-governmental organisations (NGO), or trust hospital. NGO and trust hospitals were included in the private sector category due to their modest number.

#### Outcome variable

The outcome variable is neonatal death, defined as deaths occurring within the 28 days of life (0–28 days).

#### Matching variables

Matching covariates includes maternal age (in completed years), maternal education (in completed years), birth order, household wealth quintile, caste or tribe (Scheduled caste/Scheduled tribes, other backward caste, and others), religion (Hindu/Muslims and Others), and location of residence.

### Method

To examine the relation between neonatal death and place of delivery, we first estimated neonatal mortality by place of delivery at the state and national levels. We calculated the neonatal mortality rate using the DHS methodology [13]. Further, we compared the ’nearest neighbour’ propensity score of as given neonatal death delivered in a private hospital to the closest propensity score of a neonatal death delivered in a public hospital.

Even though, it is difficult to evaluate the relationship between birthplace and neonatal deaths using cross-sectional data because women who deliver in a private institution typically differ from those who deliver in a public institution in a set of unobserved characteristics (such as belief, attitudes) and observed characteristics (socioeconomic and demographic state). Propensity score matching technique can be used to account for this. Propensity score matching eliminates most of the bias attributable to observable covariates. The difference in mean outcomes in the matched samples can be used to obtain an estimate of the average treatment effect on the treated women. Average treatment effect on the treated (ATT), Average treatment effect on the untreated (ATU) and Average treatment effect (ATE), show the estimates after matching.

### Ethics statement

The fourth round of NFHS data was used for this investigation. The NFHS was overseen scientifically and administratively by the International Institute for Population Sciences (IIPS) in Mumbai, India. IIPS is affiliated with the Government of India’s Ministry of Health and Family Welfare. Because the data were analysed anonymously using publicly accessible secondary data, no ethics approval was necessary for this work.

## Results

### Place of delivery

Table 1 presents information on India and the state-wise proportion of delivery according to home, public health facility and private health facility in five years in third to fifth column from left. At the time of the survey in India, 21 percent of births were delivered at home, 79 percent in an institute; this includes 52 percent of delivery in public hospitals. Deliveries in private hospitals range from highest in Kerala and Telangana (61.5%), followed by Gujarat (56%) and lowest in Jammu and Kashmir (7.5%) followed by Odisha (9.5%). On the other hand, deliveries in public hospitals were highest in Jammu and Kashmir (78%), followed by Odisha (76%) and lowest in Gujarat (32%). In Nagaland state proportion of home, delivery was highest, which was 67% and lowest in Telangana and Andhra Pradesh (about 8%).

**Table 1:** Proportion of live births and distribution of neonatal and infant mortality rates (per thousand) by place of delivery according to India and states, NFHS-4.

States/UT	Total N	Home delivery	Public Hospital	Private Hospital	Neonatal Mortality				Infant Mortality			
					All	Home	Public	Private	All	Home	Public	Private
India	259469	21.1	52.1	26.84	29.5	40.5	26.9	25.7	40.7	57.0	38.0	33.0
Andhra Pradesh	3127	8.5	38.3	53.3	22.3	44.7	20.9	19.8	32.9	61.2	37.9	25
Assam	10304	29.4	60	10.7	32.9	43.1	31.2	14.2	47.8	67.1	42.2	25.9
Bihar	25428	36.2	47.7	16.2	36.7	39.4	30.6	48.8	48.3	53.6	41.4	56.4
Chandigarh	194	8.4	72.4	19.3	30.5	121.1	28.1	0.0	36.3	121.1	28.1	30.4
Chhattisgarh	9281	29.8	55.9	14.4	42.1	53.6	34.9	45.6	53.9	72.3	44.8	50
Delhi	1577	15.5	55.6	28.9	16.5	34.3	15.6	8.5	29.8	75.8	27.4	8.8
Goa	416	3.1	58.2	38.7	13.3	0	13	14.8	13.3	0.0	13.0	14.8
Gujarat	7713	11.3	32.6	56.1	26.8	33.2	25.1	26.5	33.8	47.9	29.3	33.5
Haryana	7878	19.6	52.0	28.4	21.9	27.6	20.9	19.6	32.5	45	29.7	28.9
Himachal Pradesh	2928	23.6	61.6	14.8	24.2	24.6	22.7	29.8	33.6	39.8	30.1	38.0
Jammu & Kashmir	8240	14.3	78.1	7.5	23.1	27.2	23.1	15.4	32.6	34.4	33.7	17.9
Jharkhand	12201	37.79	41.81	20.43	33	41.3	27.2	29	44.1	56.1	35.6	38.9
Karnataka	12201	38.1	41.8	20.1	18.8	38.2	19	14.8	27.5	62.6	27.6	20.6
Kerala	2460	0.1	38.4	61.5	4.5	301.3	5.8	3.0	5.6	334.5	7.5	3.5
Maharashtra	9400	9.7	48.9	41.4	16.2	11.9	18	15.1	23.6	28.6	25.6	19.9
Madhya Pradesh	24588	19.2	69.5	11.4	36.7	50.8	33.1	34.9	50.8	69.5	46.9	43.3
Odisha	11091	14.6	75.9	9.5	28.3	44.2	26.4	18.3	39.4	63.9	37.0	21.0
Punjab	5216	9.5	51.7	38.8	21.6	29.4	19.9	22.1	30.2	38.8	29.7	28.6
Rajasthan	16830	16.0	63.5	20.5	29.7	27.1	29.6	31.9	41.2	41.5	41.1	41.4
Tamil Nadu	7918	1.1	66.7	32.3	66.7	32.3	16.3	8.8	20	68.5	23.3	11.5
Uttar Pradesh	41747	32.2	44.5	23.3	44.4	45.7	39.7	51.6	62.7	66.2	57.7	67.1
West Bengal	5323	24.8	56.6	18.6	22.4	37.3	20.5	8.4	28.4	42	27.7	12.5
Uttarakhand	5823	31.4	43.8	24.9	27.2	40.0	23.5	17.6	39.3	51	37.0	28.4

### Distribution of neonatal mortality rates by place of delivery

Table 1 column 6^th^ to 13^th^ depicts the distribution of neonatal mortality rates by place of delivery. Overall Neonatal mortality rate (NMR) in India is about 30 per thousand which is 10 percent higher among births delivered at home. In Uttar Pradesh, the neonatal mortality is nearly 45 per thousand, followed by Chhattisgarh (42 percent), the highest among the states. Besides these two states, overall neonatal mortality is higher than the national average among states, namely Assam, Bihar, Jharkhand and Madhya Pradesh.

Given the place of delivery, significantly high neonatal mortality was found in private health facility compared to public health facility, in Uttar Pradesh (NMR _private hospital_ = 52 Vs NMR _public hospital_ = 40), Bihar (NMR _private hospital_ = 49 Vs NMR _public hospital_ = 31) and Chhattisgarh (NMR _private hospital_ = 46 Vs NMR _public hospital_ = 35) states. In Assam and West Bengal public health facilities have high neonatal mortality relative to private hospitals. It is surprising to see that in Bihar, Himanchal Pradesh, Maharashtra, Rajasthan and Uttar Pradesh, home deliveries had lower infant mortality rates than those in private hospitals. Maharashtra and Rajasthan are the two states where NMR in public health facility is higher than home NMR.

### Predictors of choice of private health facility delivery

To show the predictors of private health facility we applied multivariable logistic regression.

The inclination towards private hospital deliveries is noticeable among women residing in the southern states of Andhra Pradesh, Tamil Nadu, and Kerala (table not shown). With the rising purchasing power of the population, there is a progressive increase in private hospital deliveries, a trend observed not only in the southern states but also in the state of Gujarat. Public hospital delivery is the preferred choice for the population in Assam, as well as in other states like Delhi, Goa, Haryana, Bihar, and Chhattisgarh. Moreover, a higher wealth index is associated with a greater preference for private institution deliveries over public settings. This trend holds true for Assam, Delhi, Goa, and Bihar, while there is insufficient evidence to draw conclusions for Haryana and Chhattisgarh.

When analyzing the birth order of women in relation to the place of delivery, it becomes evident that a significant number of women prefer private hospitals for their first delivery, while a shift towards public hospital deliveries occurs with higher parity. This trend is observed in Andhra Pradesh, Assam, Delhi, Goa, Gujarat, Haryana, and Tamil Nadu. On the other hand, in states like Bihar, Uttar Pradesh, West Bengal, Karnataka, Maharashtra, Uttarakhand, and Chhattisgarh, there is a prevailing preference for public institution deliveries regardless of the birth order. There is noticeable disparity in the choice of birthplace based on religious affiliations among women in different states. In Andhra Pradesh and Gujarat, the Muslim and other religious communities tend to prefer government hospital deliveries over Hindus. Conversely, in Delhi, Goa, and Haryana, Hindu and other religious groups exhibit a greater interest in private hospital deliveries, while Muslims in these states show a stronger preference for public hospital deliveries. Notably, in Bihar and Jammu & Kashmir, Muslim women lean towards private institution deliveries rather than public settings.

The logistic regression analysis, (table not shown), reveals that the adjusted odds ratio for neonatal deaths in India is 1.47 (95 percent CI: 1.38–1.577). This indicates that private health facilities experience significantly higher rates of neonatal deaths compared to public facilities, even after considering other important covariates in the model.

### States with effective public hospital delivery after balancing socioeconomic characteristics

#### Propensity score estimates: Effect of place of delivery on neonatal mortality

Table 2 presents propensity score estimates that examine the impact of the place of delivery on neonatal deaths. Initially, a notable difference in neonatal mortality (above one percent) between private and public institutional deliveries was observed in Himachal Pradesh, Uttar Pradesh, West Bengal, and Assam without any matching. However, after matching, significant differences between public and private institutional deliveries were found in Haryana, Rajasthan, Uttarakhand, Madhya Pradesh, Uttar Pradesh, Bihar, Jharkhand, West Bengal, Assam, and Gujarat. On the other hand, Punjab, Chhattisgarh, Goa, and Andhra Pradesh showed a more modest difference (between 0.5 to 1 percent) in neonatal deaths between private and public institutional deliveries after matching. Among the states with higher neonatal mortality in the private sector, Rajasthan initially exhibited only a 0.20 percent difference in neonatal deaths between public and private hospitals in the unmatched sample. However, after matching for socioeconomic and demographic covariates, the analysis revealed that neonatal mortality in private hospitals is 3.2 percent higher compared to public hospitals.

**Table 2:** Description of the estimated propensity scores to show the effect of place of delivery on neonatal deaths across selected states in India, 2015–16.

States	Estimates After Matching		Estimates Before matching	
	Private Health Facility	Public Health Facility	Private Health Facility	Public Health Facility
**Northern Region**				
Delhi	0.0091	0.0136	0.0091	0.0172
Haryana	0.019	0.030	0.019	0.021
Himachal Pradesh	0.021	0.024	0.021	0.009
Jammu & Kashmir	0.016	0.016	0.016	0.022
Punjab	0.025	0.020	0.025	0.015
Rajasthan	0.032	0.000	0.032	0.030
Uttarakhand	0.018	0.030	0.018	0.024
**Central Region**				
Chhattisgarh	0.046	0.041	0.046	0.036
Madhya Pradesh	0.032	0.013	0.032	0.032
Uttar Pradesh	0.053	0.069	0.053	0.038
**Eastern Region**				
Bihar	0.051	0.036	0.051	0.031
Jharkhand	0.025	0.007	0.025	0.027
Odisha	0.022	0.019	0.022	0.029
West Bengal	0.007	0.020	0.007	0.021
**North East Region**				
Assam	0.014	0.033	0.014	0.030
**Western Region**				
Goa	0.013	0.007	0.013	0.014
Gujarat	0.028	0.046	0.028	0.020
Maharashtra	0.019	0.016	0.019	0.018
**Southern Region**				
Andhra Pradesh	0.023	0.015	0.023	0.023
Karnataka	0.019	0.016	0.019	0.019
Tamil Nadu	0.010	0.010	0.010	0.016

In Madhya Pradesh, the initial unmatched sample did not reveal any significant difference in neonatal mortality between public and private hospitals. However, after adjusting for relevant factors, the analysis showed that neonatal deaths for births delivered in private hospitals were 3.2 percent. Interestingly, if women with the same characteristics had delivered in public hospitals instead, the neonatal mortality rate could have reduced to 1.9 percent (ATT value for treated is 0.032, and for control is 0.013).

Similarly, in Jharkhand, the ATT value for treated (those who delivered in private health centers) was 0.026, while for control (those who would have delivered in public health facilities), it was 0.007. This indicates that if women who delivered in private health centers had chosen public health facilities instead, only 0.70 percent of them would have experienced neonatal deaths. These findings suggest that in Jharkhand state, public hospitals provide better outcomes compared to private hospitals.

In Bihar state, the initial unmatched sample estimate indicates that neonatal mortality increased by 2.0 percent when mothers gave birth in private hospitals compared to public hospitals. However, after employing nearest neighbourhood matching with the replacement method, the ATT value for treated was 0.051, while for control, it was 0.036. This suggests that neonatal deaths increased by 1.5 percent if mothers gave birth in private hospitals compared to public hospitals. Similarly, in Punjab, the unmatched sample estimate demonstrates a 0.5 percent increase in neonatal deaths if mothers gave birth in private hospitals. After using the nearest neighbor matching with the replacement method, the ATT value for treated was 0.025, and for control, it was 0.015, indicating that neonatal deaths increased by 1.0 percent if mothers chose private hospitals over public hospitals in Punjab state.

### States with effective private hospital delivery after balancing socioeconomic characteristics

It is evident from Table 2 that in states like Haryana, Himachal Pradesh, Uttarakhand, West Bengal, Uttar Pradesh, Assam and Gujrat, private hospitals have had fewer neonatal deaths compared to the public hospital in the last five years. The Haryana unmatched sample estimate shows that neonatal deaths decrease by 1.1 percent if mothers give birth in private hospitals. After matching, those births born in private hospitals were a 1.1 percent less chance of dying during the neonatal period. The propensity scores matching process balanced the socioeconomic characteristics between the public and private hospitals in Himachal Pradesh and found an opposite result from the unmatched sample, birth delivered in public hospitals were having 0.3 percent higher chance to experience neonatal deaths compared to birth delivered in private hospitals.

For Uttarakhand, the unmatched sample estimate indicates a 0.6 percent difference in neonatal deaths between public and private health facilities. However, after implementing matching, the estimate reveals that births born in private hospitals had a 1.2 percent lower chance of dying during the neonatal period compared to those born in public hospitals. Similarly, West Bengal state exhibits a similar pattern, where conditions in public health facilities were worse relative to private health care. In Uttar Pradesh, the unmatched sample revealed that neonatal mortality in private hospitals was 1.6 percent higher than in public hospitals. Surprisingly, after implementing matching, the results showed that births born in private hospitals had a 1.6 percent lower chance of dying during the neonatal period compared to those born in public hospitals. In Assam, both before and after matching, the results demonstrated fewer neonatal deaths in private facilities than in public institutions. For Gujarat state, the unmatched sample estimate indicated that births delivered in public hospitals had a lower chance of neonatal deaths. However, after matching, the results showed that those births delivered in public hospitals had a 1.8 percent higher chance of dying during the neonatal period compared to private hospital deliveries.

### Antenatal and postpartum care received in the last delivery by place of delivery

The state-specific PSM analysis reveals variations in the performance of public and private health facilities concerning pregnancy and child care. However, it is important to note that the PSM analysis is based on all births that occurred during the last five years, while information on antenatal and postnatal care is only available for recent births. Due to this limitation, we were unable to match these variables in the PSM analysis.

To effectively assess the quality of both public and private hospitals, it becomes crucial to determine whether women received all essential antenatal and postnatal care services. Neonatal death is closely associated with the quality of antenatal and postnatal care provided, making this assessment particularly important.

Furthermore, it is vital to investigate whether the place where women received antenatal and postnatal care is the same as the place of delivery. There is a possibility that some mothers received all ANC services from a private health facility but opted to deliver in a public hospital, or vice versa. Addressing this aspect is important and therefore the first two columns in S1 Table display the percentage of mothers who delivered in public and private hospitals and out of them the percentage of those who received antenatal care at the same place where they delivered.

We also analysed a number of metrics related to prenatal and postnatal care (S1 Table shows the percentage distribution of women received various antenatal care services at public and private facility according to selected states in India, NFHS-2015-16 and S2 Table shows the percentage distribution of women received various post delivery services at public and private facility according to selected states in India, NFHS-2015-16) to get a sense of the standard of care provided to women by public and private hospitals. Understanding these patterns is essential for making comprehensive assessments of the performance and quality of public and private health facilities in maternal and child health services.

Initially, we examined the coherence in location selection for both ANC utilization and delivery. The findings reveal that across almost all states, a greater proportion of women who delivered their babies in private hospitals preferred to seek antenatal care at private hospitals, while a higher percentage of women who gave birth in public hospitals opted for antenatal care at public hospitals. For Antenatal Care, we considered factors such as ’information provided about pregnancy complications,’ ’blood pressure monitoring,’ and ’urine sample collection.’ As for post-delivery care, we assessed the ’average hospital stay after C-section and vaginal delivery,’ ’postpartum check-up before discharge,’ ’initiation of breastfeeding within the first hour,’ and the ’initial check-up within an hour after delivery.’

The findings highlight that Andhra Pradesh, Bihar, Chhattisgarh, Goa, Jharkhand, Karnataka, Madhya Pradesh, Maharashtra, Odisha, Punjab, and Rajasthan exhibit the highest rates of newborn fatalities in private health facilities. Specifically, in Andhra Pradesh, women receiving ANC in private health facilities are less likely to be informed about pregnancy complications, undergo regular blood pressure and urine tests, spend fewer days in hospitals after childbirth, initiate breastfeeding within the first hour after delivery, and have their first check-up within an hour, compared to those giving birth in public hospitals.

Compared to private health facilities in Bihar, a lower proportion of women breastfed within the first hour. Similarly, in Chhattisgarh’s private hospitals, the average duration of post-delivery care for new mothers, the rate of breastfeeding initiation within the first hour, and the frequency of the first check-up within an hour were all lower than those in public hospitals.

In the states of Karnataka, Madhya Pradesh, Maharashtra, Odisha, Punjab, and Rajasthan, the average days spent in the hospital after delivery and the rate of breastfeeding initiation within the first hour were lower in private hospital deliveries when compared to public hospital deliveries.

Conversely, in states with higher neonatal mortality rates in public hospitals, women were less likely to receive information about pregnancy complications, had fewer instances of blood pressure measurement, and a lower proportion of women reported having their first check-up within an hour after delivery compared to women who gave birth in private hospitals.

The evaluation of states based on the continuum of care performance by public and private healthcare institutions, which includes measures like blood pressure and urine sample collection, information gathering on pregnancy complications, and postpartum check-ups before discharge, indicates that private hospitals have generally shown better performance compared to public hospitals. However, it is noteworthy that across almost all states, a higher percentage of women reported breastfeeding their infants within an hour of birth in public hospitals as compared to private ones.

## Discussion

In India, it is often assumed that the quality of care offered in the private sector is superior, competent, and responsible to that provided in the public sector [14]. In light of this assumption, the current study explores the association between birthplaces and neonatal deaths in India. Contrary to the mentioned assumption, the present study’s PSM analysis showed a substantial difference in neonatal mortality in India’s public and private health care. At national level, private health facilities have higher odds of early neonatal death than public.

A recent study focusing on health services in Lower-Middle-Income Countries (LMIC), including India, emphasizes that the quality of healthcare delivery plays a more significant role in newborn fatalities than the non-utilization of services [9]. Associated challenges, such as inconsistent management of complex pregnancies and newborn cases due to inadequate infrastructure, primarily impact essential neonatal care at healthcare facilities [15, 16]. Additionally, the higher neonatal mortality rate in private facilities may be linked to last-minute obstetric referrals to better-equipped facilities, particularly from public to private settings, stemming from factors like small case sizes, facility overcrowding, limited human resources, and early discharges [17].

Conversely, recent research suggests that the quality of public health facilities has shown improvement in terms of physical infrastructure, the availability of essential equipment, enhanced accessibility, increased staff appointments, notably auxiliary nurse midwives (ANM), and overall service delivery strengthening, facilitated by increased funding under the National Health Mission (NHM) over the past two decades [18, 19].

In this study, Propensity Score Matching (PSM) was utilized for all major states to assess the influence of the place of delivery on neonatal deaths. Prior to matching socioeconomic and demographic variables, newborn mortality rates were found to be higher for deliveries in public hospitals in approximately half of the states, while in the remaining states, neonatal mortality was higher for deliveries in private hospitals. The results obtained from the matched sample of most states contradict the common perception that the quality of care in public health facilities is always worse than that in private facilities [20]. This perception stems from the belief that private health facilities, being diverse and larger, have the capacity to cater to a vast population effectively, overlooking the fact that many small private facilities in rural areas are managed by semi or unqualified personnel [18]. Studies conducted in rural settings in India have revealed that such private facilities lack essential infrastructure, human resources, and backup equipment to handle critical maternal and child health emergencies [21, 22].

Despite the fact that neonatal mortality rates are higher in private hospitals, they outperform public hospitals in basic delivery care procedures, as indicated by the analysis. Previous research also suggests that the preference for private over public healthcare facilities could be attributed to the lack of available healthcare facilities when needed and the provision of poorer neonatal and obstetric care during weekends [23, 24]

In summary, the study’s findings challenge the notion of superior quality in private health facilities compared to public ones, highlighting the existing disparities and limitations in both sectors. While private hospitals may excel in certain aspects of basic delivery care, they still face significant challenges in providing adequate maternal and child health services, particularly in rural areas. Meanwhile, public health facilities have shown improvements in recent years, indicating the potential for enhanced healthcare delivery in the public sector.

In the case of maternal and child health, women or families have a level of expectation about the health services they will receive from either public or private facilities. This study’s findings show that women who had ANC services in private hospitals prefer private hospitals for delivery, and the same pattern is visible in women who choose public hospitals. In other words, findings show that, in terms of place of delivery, women choose familiarity and support. This might be due to the excellent guidance offered and the rapport built between the mother and the healthcare personnel during antenatal care visits, this has a considerable favourable effect on lowering neonatal mortality [25]. Accessible, available, affordable and quality healthcare should be ensured by public institutions so that mothers opt for more public institutions [26].

A recent study pointed out that the market share of private hospitals in maternal and child care is increasing in LMICs [27]. Making this study has a crucial implication, particularly in ensuring quality maternal and child health, the policymakers and researchers should focus on quality improvement overall, with a particular focus private sector. In states where neonatal mortality is higher among private facilities, Priority should be given to addressing the issues of understaffing, improving emergency care and referral services, and alleviating facility overload.

To increase accountability, better inspection, licensing and quality control of private sector facilities and improve the quality of health services, PPP (Public-Private Partnership) in providing maternal child care in areas where the imbalance is found will be a more remarkable step.

The strong suit of this study is that the data provide comprehensive information about the study population obtained from a comprehensive nationally-representative sample, improving external validity and generalisation. However, the current study’s findings need to be interpreted with limitations. Firstly, the data is also from a cross-sectional survey, which limits the scope of establishing causality among variables. Secondly, the causes of neonatal mortality may be due to non-institutional factors like maternal depletion, pre-term complications, and infections, etc. [28, 29]. Besides, all the findings presented in this paper are reasonable to call the attention of policymakers to system-wide improvement and more focused future research.

## Conclusion

The study’s findings stress the need for focused efforts to improve the quality of maternal and child health care in both the public and private sectors, with particular attention to addressing the higher neonatal mortality rates observed in private facilities. Policymakers and researchers should prioritize quality improvement initiatives, tackling issues such as understaffing, poor emergency care, referral services, and facility overload in private health facilities to enhance maternal and child health outcomes.

To ensure accountability and raise the standard of health services, implementing Public-Private Partnerships (PPPs) in areas with imbalances between public and private facilities is recommended. This collaborative approach can lead to better inspection, licensing, and quality control measures for private sector facilities, ensuring they meet appropriate standards.

In conclusion, the study underscores the necessity for comprehensive improvements in maternal and child health care at a system-wide level. Policymakers must strive to provide accessible, available, affordable, and high-quality healthcare in both public and private sectors to achieve better maternal and child health outcomes in India. Enhanced national health policies and increased public-private collaborations are vital steps towards achieving these goals.

## Supporting information

S1 Table Percentage distribution of women received various antenatal care services at public and private facility according to selected states in India, NFHS-2015-16. (DOCX)

S2 Table Percentage distribution of women received various post delivery services at public and private facility according to selected states in India, NFHS-2015-16. (DOCX)
