# Supplementary material for: Is the quality of public health facilities always worse compared to private health facilities: Association between birthplace on neonatal deaths in the Indian states
Source: PLoS One. 2023 Dec 27;18(12):e0296057. doi: 10.1371/journal.pone.0296057 (PMC10752527; doi:10.1371/journal.pone.0296057)
Supplement: S1 Table — (DOCX) [file pone.0296057.s001.docx]

Supplementary Table – S1 Percentage distribution of women received various antenatal care services at public and private facility according to selected states in India, NFHS-2015-16.

| **State** | **Delivered in Public Hospital** | | **Delivered in Private Hospital** | | **Told about pregnancy complications** | | | | **Blood Pressure taken** | | | | **Urine Sample Taken** | | | |
| --- | --- | --- | --- | --- | --- | --- | --- | --- | --- | --- | --- | --- | --- | --- | --- | --- |
|  | **ANC in**  **Public** | **ANC in**  **Private** | **ANC in**  **Public** | **ANC in Private** | **Public** | | **Private** | | **Public** | | **Private** | | **Public** | | **Private** | |
|  | **%** | **%** | **%** | **%** | **N** | **%** | **N** | **%** | **N** | **%** | **N** | **%** | **N** | **%** | **N** | **%** |
| **Andhra Pradesh** | 55 | 42.8 | 12.0 | 85.1 | 609 | 51.7 | 842 | 50.4 | 868 | 73.7 | 1,190 | 71.3 | 866 | 73.5 | 1,183 | 70.8 |
| **Assam** | 76 | 6.0 | 29.0 | 53.9 | 3815 | 61.0 | 694 | 70.9 | 4,926 | 78.8 | 858 | 87.6 | 4,596 | 73.5 | 831 | 84.9 |
| **Bihar** | 56 | 23.2 | 14.0 | 67.3 | 2545 | 20.2 | 1289 | 30.7 | 3,653 | 29.0 | 2,064 | 49.1 | 3,479 | 27.6 | 2,023 | 48.2 |
| **Chandigarh** | 41.9 | 6.0 | 23.0 | 25.8 | 87 | 62.1 | 29 | 80.6 | 104 | 74.3 | 31 | 86.1 | 104 | 74.3 | 31 | 86.1 |
| **Chhattisgarh** | 77.82 | 10.3 | 26.0 | 62.2 | 2883 | 55.3 | 765 | 60.1 | 3,669 | 70.4 | 999 | 78.5 | 3,493 | 67.0 | 967 | 76.0 |
| **Delhi** | 69.15 | 11.0 | 14.0 | 71.5 | 400 | 43.1 | 217 | 49.3 | 678 | 73.0 | 345 | 78.4 | 669 | 72.0 | 339 | 77.0 |
| **Goa** | 75.63 | 8.0 | 12.0 | 68.2 | 162 | 65.3 | 116 | 73.9 | 195 | 78.6 | 135 | 86.0 | 196 | 79.0 | 134 | 85.4 |
| **Gujarat** | 64 | 14.0 | 15.0 | 65.6 | 1213 | 45.0 | 2038 | 51.5 | 1,615 | 59.9 | 2,696 | 68.1 | 1,548 | 57.4 | 2,601 | 65.7 |
| **Haryana** | 66 | 18.0 | 22.0 | 63.6 | 2237 | 54.2 | 1235 | 56.4 | 2,641 | 64.0 | 1,486 | 67.8 | 2,580 | 62.5 | 1,453 | 66.3 |
| **Himachal Pradesh** | 58 | 4.0 | 20.0 | 37.7 | 878 | 49.5 | 254 | 59.1 | 1,285 | 72.5 | 339 | 78.8 | 1,302 | 73.4 | 330 | 76.7 |
| **Jammu and Kashmir** | 88.87 | 9.0 | 23.0 | 74.5 | 2934 | 46.4 | 302 | 61.5 | 4,500 | 71.2 | 402 | 81.9 | 4,550 | 72.0 | 399 | 81.3 |
| **Jharkhand** | 64.48 | 27.0 | 17.0 | 73.9 | 1577 | 30.0 | 851 | 36.7 | 2,816 | 53.5 | 1,567 | 67.6 | 2,591 | 49.2 | 1,536 | 66.2 |
| **Karnataka** | 65.11 | 13.0 | 18.0 | 64.1 | 2412 | 48.8 | 1180 | 50.4 | 3,361 | 68.0 | 1,724 | 73.6 | 3,358 | 67.9 | 1,719 | 73.4 |
| **Kerala** | 48.05 | 3.5 | 1.0 | 50.6 | 406 | 42.3 | 583 | 38.9 | 838 | 87.3 | 1,256 | 83.9 | 841 | 87.6 | 1,255 | 83.8 |
| **Madhya Pradesh** | 65 | 13.0 | 19.0 | 58.3 | 6485 | 38.3 | 1407 | 53.9 | 8,877 | 52.4 | 1,879 | 71.9 | 8,583 | 50.7 | 1,868 | 71.5 |
| **Maharashtra** | 48 | 13.0 | 9.0 | 50.2 | 2265 | 47.7 | 1844 | 50.8 | 3,302 | 69.5 | 2,648 | 73.0 | 3,301 | 69.5 | 2,644 | 72.9 |
| **Odisha** | 73.78 | 9.0 | 31.9 | 52.1 | 4975 | 59.3 | 664 | 67.3 | 6,404 | 76.3 | 864 | 87.6 | 6,217 | 74.1 | 830 | 84.2 |
| **Punjab** | 48 | 8.0 | 11.0 | 50.0 | 1947 | 68.4 | 1384 | 71.7 | 2,146 | 75.4 | 1,557 | 80.7 | 2,131 | 74.9 | 1,534 | 79.5 |
| **Rajasthan** | 72 | 22.6 | 26.0 | 66.8 | 4099 | 37.8 | 1490 | 45.3 | 6,329 | 58.4 | 2,222 | 67.6 | 6,202 | 57.2 | 2,199 | 66.9 |
| **Tamil Nadu** | 67 | 9.0 | 17.0 | 61.8 | 3253 | 61.2 | 1587 | 63.1 | 3,745 | 70.4 | 1,873 | 74.5 | 3,740 | 70.3 | 1,871 | 74.4 |
| **Uttar Pradesh** | 61 | 21.0 | 21.0 | 66.4 | 4424 | 24.3 | 3401 | 33.6 | 6,347 | 34.8 | 5,473 | 54.1 | 6,661 | 36.6 | 5,227 | 51.7 |
| **Uttarakhand** | 67.08 | 11.0 | 23.0 | 59.3 | 938 | 34.6 | 501 | 40.8 | 1,551 | 57.1 | 798 | 64.9 | 1,515 | 55.8 | 751 | 61.1 |
| **West Bengal** | 35.03 | 15.4 | 10.6 | 35.0 | 1835 | 59.0 | 625 | 70.1 | 2,429 | 78.1 | 776 | 87.0 | 2,368 | 76.2 | 771 | 86.4 |
