# Supplementary material for: Is the quality of public health facilities always worse compared to private health facilities: Association between birthplace on neonatal deaths in the Indian states
Source: PLoS One. 2023 Dec 27;18(12):e0296057. doi: 10.1371/journal.pone.0296057 (PMC10752527; doi:10.1371/journal.pone.0296057)
Supplement: S2 Table — (DOCX) [file pone.0296057.s002.docx]

Supplementary Table – S2 Percentage distribution of women received various post delivery services at public and private facility according to selected states in India, NFHS-2015-16.

| **State** | **Average Days spent in hospital post-delivery (C-section Delivery)** | | **Average Days spent in hospital post- delivery (Vaginal Delivery)** | | **Postpartum check-up before discharge** | | | | **Breastfeeding within the 1st hour** | | **1st check-up after delivery in an hour** | | |
| --- | --- | --- | --- | --- | --- | --- | --- | --- | --- | --- | --- | --- | --- |
|  | **Public Hospital** | **Private Hospital** | **Public Hospital** | **Private Hospital** | **Public** | | **Private** | | **Public** | **Private** | **Public** | **Private** |  |
|  |  |  |  |  | **N** | **%** | **N** | **%** | **%** | **%** | **%** | **%** |  |
| **Andhra Pradesh** | 13.3 | 10.4 | 3.1 | 2.4 | 770 | 65.4 | 1,029 | 61.6 | 78.5 | 65.5 | 51.8 | 49.8 |  |
| **Assam** | 8.1 | 6.6 | 2.2 | 2.8 | 3,887 | 62.2 | 776 | 79.3 | 77.7 | 70.6 | 42.7 | 58.9 |  |
| **Bihar** | 9.7 | 12.0 | 0.9 | 1.5 | 5,131 | 40.7 | 2,318 | 55.2 | 68.3 | 49.7 | 24.6 | 31.4 |  |
| **Chandigarh** | 9.4 | 8.0 | 2.9 | 1.9 | 102 | 72.9 | 32 | 88.9 | 63.6 | 38.7 | 50.9 | 75.0 |  |
| **Chhattisgarh** | 7.1 | 6.8 | 2.2 | 2.7 | 3,000 | 57.6 | 905 | 71.1 | 81.8 | 60.1 | 37.4 | 50.1 |  |
| **Delhi** | 10.3 | 9.7 | 5.1 | 9.1 | 505 | 54.4 | 287 | 65.2 | 86.6 | 77.7 | 64.1 | 70.9 |  |
| **Goa** | 6.2 | 4.3 | 2.3 | 1.6 | 186 | 75.0 | 129 | 82.2 | 76.1 | 69.5 | 30.4 | 35.3 |  |
| **Gujarat** | 6.9 | 5.8 | 1.4 | 1.4 | 1,326 | 49.2 | 2,219 | 56.0 | 74.5 | 60.2 | 37.2 | 43.3 |  |
| **Haryana** | 5.9 | 5.7 | 2.2 | 1.9 | 2,343 | 56.8 | 1,390 | 63.4 | 61.1 | 47.7 | 36.5 | 41.6 |  |
| **Himachal Pradesh** | 6.3 | 3.9 | 2.0 | 2.2 | 1,221 | 68.9 | 332 | 77.2 | 64.4 | 55.9 | 37.5 | 48.6 |  |
| **Jammu and Kashmir** | 9.3 | 8.1 | 1.5 | 1.7 | 4,219 | 66.8 | 369 | 75.2 | 74.5 | 53.3 | 26.0 | 38.6 |  |
| **Jharkhand** | 11.9 | 12.7 | 3.1 | 4.5 | 2,221 | 42.2 | 1,389 | 59.9 | 75.3 | 72.1 | 36.9 | 42.7 |  |
| **Karnataka** | 7.4 | 6.8 | 5.5 | 4.1 | 2,333 | 47.2 | 1,253 | 53.5 | 83.8 | 79.8 | 61.1 | 58.2 |  |
| **Kerala** | 9.8 | 9.3 | 2.7 | 2.8 | 762 | 79.4 | 1,150 | 76.8 | 69.2 | 53.4 | 24.8 | 40.4 |  |
| **Madhya Pradesh** | 11.6 | 10.7 | 3.0 | 3.1 | 7,722 | 45.6 | 1,704 | 65.2 | 83.6 | 73.9 | 42.7 | 47.2 |  |
| **Maharashtra** | 7.4 | 5.3 | 2.7 | 2.3 | 2,913 | 61.3 | 2,391 | 65.9 | 67.6 | 39.7 | 27.7 | 32.3 |  |
| **Odisha** | 12.4 | 11.2 | 2.1 | 3.4 | 5,642 | 67.3 | 823 | 83.5 | 86.8 | 80.6 | 46.6 | 57.2 |  |
| **Punjab** | 6.2 | 5.8 | 2.0 | 1.9 | 2,048 | 72.0 | 1,473 | 76.4 | 64.2 | 48.5 | 49.1 | 55.6 |  |
| **Rajasthan** | 8.1 | 6.5 | 2.5 | 1.9 | 5,662 | 52.2 | 2,000 | 60.8 | 62.7 | 52.2 | 28.9 | 37.9 |  |
| **Tamil Nadu** | 12.4 | 9.4 | 5.4 | 4.6 | 3,536 | 66.5 | 1,858 | 73.9 | 81.4 | 71.5 | 37.3 | 41.8 |  |
| **Uttar Pradesh** | 8.9 | 7.8 | 1.1 | 1.3 | 8,797 | 48.3 | 6,274 | 62.0 | 57.1 | 35.9 | 34.3 | 45.6 |  |
| **Uttarakhand** | 10.4 | 7.6 | 1.7 | 1.8 | 1,481 | 54.6 | 807 | 65.7 | 64.2 | 38.7 | 35.7 | 39.9 |  |
| **West Bengal** | 8.1 | 6.9 | 2.6 | 3.2 | 1,921 | 61.8 | 687 | 77.0 | 71.7 | 58.4 | 35.1 | 51.4 |  |
